# Epigenetic inheritance of diet-induced and sperm-borne mitochondrial RNAs

**DOI:** 10.1038/s41586-024-07472-3

**Published:** 2024-06-05

**Authors:** A. Tomar, M. Gomez-Velazquez, R. Gerlini, G. Comas-Armangué, L. Makharadze, T. Kolbe, A. Boersma, M. Dahlhoff, J. P. Burgstaller, M. Lassi, J. Darr, J. Toppari, H. Virtanen, A. Kühnapfel, M. Scholz, K. Landgraf, W. Kiess, M. Vogel, V. Gailus-Durner, H. Fuchs, S. Marschall, M. Hrabě de Angelis, N. Kotaja, A. Körner, R. Teperino

**Affiliations:** 1 Institute of Experimental Genetics, Helmholtz Zentrum München, German Research Center for Environmental Health (GmbH), Neuherberg, Germany; 2 German Center for Diabetes Research (DZD), Neuherberg, Germany; 3 Unit of in vivo and in vitro Models, Center for Biological Sciences, Department of Biological Sciences and Pathobiology, University of Veterinary Medicine Vienna, Vienna, Austria; 4 IFA-Tulln, University of Natural Resources and Life Sciences, Vienna, Austria; 5 Institute of Animal Breeding and Genetics, Department of Biological Sciences and Pathobiology, University of Veterinary Medicine Vienna, Vienna, Austria; 6 Group Molecular Reproduction, IFA-Tulln, Tulln, Austria; 7 Institute of Biomedicine, Integrative Physiology and Pharmacology Unit, University of Turku, Turku, Finland; 8 Center for Population Health Research, University of Turku and Turku University Hospital, Turku, Finland; 9 University of Leipzig, Medical Faculty, Institute for Medical Informatics, Statistics and Epidemiology, Leipzig, Germany; 10 Center for Pediatric Research Leipzig (CPL), Hospital for Children & Adolescents, University of Leipzig, Leipzig, Germany; 11 LIFE Leipzig Research Center for Civilization Diseases, University of Leipzig, Leipzig, Germany; 12 German Mouse Clinic, Helmholtz Zentrum München, German Research Center for Environmental Health (GmbH), Neuherberg, Germany; 13 Chair of Experimental Genetics, TUM School of Life Sciences, Technische Universität München, Freising, Germany; 14 Helmholtz Institute for Metabolic Obesity and Vascular Research (HI-MAG), Helmholtz Zentrum München at the University of Leipzig and University Hospital Leipzig, Leipzig, Germany

**Keywords:** Energy metabolism, Epigenetics

## Abstract

Spermatozoa harbour a complex and environment-sensitive pool of small non-coding RNAs (sncRNAs)^1^, which influences offspring development and adult phenotypes^1–7^. Whether spermatozoa in the epididymis are directly susceptible to environmental cues is not fully understood^8^. Here we used two distinct paradigms of preconception acute high-fat diet to dissect epididymal versus testicular contributions to the sperm sncRNA pool and offspring health. We show that epididymal spermatozoa, but not developing germ cells, are sensitive to the environment and identify mitochondrial tRNAs (mt-tRNAs) and their fragments (mt-tsRNAs) as sperm-borne factors. In humans, mt-tsRNAs in spermatozoa correlate with body mass index, and paternal overweight at conception doubles offspring obesity risk and compromises metabolic health. Sperm sncRNA sequencing of mice mutant for genes involved in mitochondrial function, and metabolic phenotyping of their wild-type offspring, suggest that the upregulation of mt-tsRNAs is downstream of mitochondrial dysfunction. Single-embryo transcriptomics of genetically hybrid two-cell embryos demonstrated sperm-to-oocyte transfer of mt-tRNAs at fertilization and suggested their involvement in the control of early-embryo transcription. Our study supports the importance of paternal health at conception for offspring metabolism, shows that mt-tRNAs are diet-induced and sperm-borne and demonstrates, in a physiological setting, father-to-offspring transfer of sperm mitochondrial RNAs at fertilization.

## Abstract (web summary)

A study shows that epididymal spermatozoa are sensitive to preconception diet, identifies mitochondrial tRNAs and their fragments as sperm-borne factors and demonstrates epigenetic inheritance of mitochondrial tRNAs.

## Main

Apart from Mendelian inheritance, fathers use alternative routes for intergenerational information transfer. One of these is a complex, dynamic and environment-sensitive pool of sncRNAs that are stored in mature spermatozoa^1^, delivered to the oocytes at fertilization^9^, and influence embryonic development^10,11^ and adult phenotypes^1–7,11^.

Production of mature haploid spermatozoa from spermatogonial stem cells is a two-step process consisting of spermatogenesis in the seminiferous tubules (about 35 days in the mouse), followed by maturation in the epididymis (about 7 days in the mouse)^12^. Both phases constitute potential windows of environmental susceptibility for the sperm epigenome^8^. The current accepted model is that the sperm sncRNA pool is modified during the epididymal transit with substantial contribution from the epididymal epithelial cells^4,10,11,13–16^ and, therefore, environmental perturbations are thought to act primarily on the epididymis. On the other hand, despite the presence of the blood–testis barrier^17^, perturbations targeted to spermatogenesis have intergenerational or even transgenerational consequences^18,19^. We focused on the intergenerational sequelae of paternal overweight and attempted to dissect the relative contributions of testicular and epididymal exposures. The studies in the current body of literature used a patchwork of different exposures to a standard high-fat diet (HFD; 60% fat)—from 6 to 22 weeks—alongside a wide range of starting ages—from 4 to 9 weeks of age (for example, refs. ^5,20–25^). These studies do not account for spermatogenesis timing; report combined testicular and epididymal effects on the sperm epigenome; and are not suitable to study susceptibility windows and identify environmental sensors in the male reproductive tract.

Our results show that paternal preconceptional exposure to 2 weeks of HFD at 6 weeks of age, when the first wave of spermatogenesis is completed and the produced sperm is undergoing maturation in the epididymis^26^, induces partial penetrant glucose intolerance and insulin resistance in male offspring. Of note, the same exposure does not affect the developing germ cells in the testis and spermatogenesis does not contribute to the paternal intergenerational effects. Mechanistically, we found that mitochondrial-encoded tRNAs (mt-tRNAs) and their fragments (mt-tsRNAs) are dynamically regulated by the HFD challenge. Our data indicate a sperm-borne origin of these sncRNAs, in keeping with their enrichment in epididymal spermatozoa compared to epididymosomes and sperm-associated cytoplasmic droplets, and the reported active transcription of the mtDNA in mature spermatozoa^27^.

Two decades ago, it was shown that protamin 2 and clusterin mRNAs are transferred from spermatozoa to the oocyte at fertilization^9^, triggering the use of zygotic microinjections to study the role of sperm RNAs in paternal effects (reviewed in ref. ^28^). These experiments do not demonstrate intergenerational transfer of sperm RNAs, but the consequences of their perturbations in early embryos. Harnessing the genetic diversity of mtDNA and single-embryo transcriptomics, we have genetically tracked the parental origin of mt-tRNAs in early embryos and discovered them to be transferred from spermatozoa at fertilization. Our data support a model by which acute HFD exposure induces mitochondrial dysfunction in somatic tissues and spermatozoa, in which it is compensated by an upregulation of mtDNA transcription. This leads to an accumulation of mt-sncRNAs and their fragments, which are epigenetically inherited and probably contribute to modifying transcription in early embryos and glucose metabolism in adult offspring. In keeping with our model, in-depth mouse phenotypic data from the International Mouse Phenotyping Consortium (IMPC)^29^ and sperm sncRNA analyses highlighted alterations of spermatozoa mt-tsRNAs downstream of genetically induced mitochondrial dysfunction and paternal non-genetic control of offspring glucose homeostasis. Last, using two independent human cohorts, we have shown that sperm mt-tsRNAs are associated with body mass index (BMI) and that paternal BMI at conception is an independent determinant of offspring metabolic health.

Altogether, the findings of our study strengthen the relevance of paternal health at conception for offspring metabolism, show that mt-tRNAs (and their fragments) are diet-induced and sperm-borne, and demonstrate, in a physiological setting, epigenetic inheritance of mt-tRNAs.

## Epididymal sperm is susceptible to diet

**Fig. 1:**
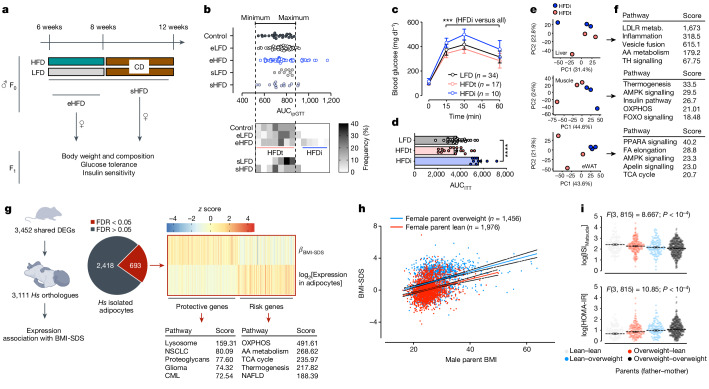
Paternal overweight at conception is important for offspring metabolism in mice and humans. **a**, Experimental design. CD, chow diet. **b**, Glucose tolerance of unexposed male offspring (F_1_) of HFD-exposed bucks. Top: AUC_ipGTT_. Bottom: frequency distribution analysis to identify tolerant and intolerant animals. *n* = 60 male mice across 4 cohorts with 5 litters each and 3 males per litter (eLFD and eHFD bucks); *n* = 10–15 (sLFD and sHFD bucks) including 1 cohort with 5 litters and 3 males per litter. **c**,**d**, Glucose tolerance (**c**; mean ± s.e.m.) and insulin sensitivity (**d**; mean ± s.e.m.) of male offspring (F_1_) of HFD-exposed bucks. Data represent a re-phenotyping of the animals in **c** carried out 8 weeks after the first phenotyping. Significance calculated by a two-way (**c**; *n* in graph) or one-way (**d**; *n* as in **c**) analysis of variance (ANOVA; ***P < 10^−4^). ITT, insulin tolerance test. **e**,**f**, PCA plot (**e**) and functional enrichment analysis (KEGG (Kyoto Encyclopedia of Genes and Genomes); **f**) of peripheral tissue RNA-seq data from HFDt and HFDi F_1_ animals. eWAT, epididymal white adipose tissue; metab., metabolism; AA, amino acid; TH, thyroid hormone; OXPHOS, oxidative phosphorylation; FA, fatty acid; TCA, tricarboxylic acid. **g**, Left: overlap between genes differentially expressed in tissues from HFDi mice and their human orthologues associated with childhood obesity. Right: functional enrichment analysis (KEGG) of the overlapping genes (*n* = 693) pre-classified as protective and risk genes for childhood obesity on the basis of the *β*-score for BMI-SDS. DEGs, differentially expressed genes; *Hs*, *Homo sapiens*; FDR, false discovery rate; NSCLC, non-small cell lung cancer; CML, chronic myeloid leukaemia; NAFLD, non-alcoholic fatty liver disease. **h**, Scatter plot of children’s body weight trajectories as a function of paternal BMI at conception in families with mothers who were lean (red line; *r* = 0.2611; *P* value < 10^−4^) or overweight (blue line; *r* = 0.3467; *P* value < 10^−4^) at conception. Significant association calculated by linear regression analysis. **i**, Insulin sensitivity, measured as ISI_Matsuda_ (top) or homeostatic model assessment for insulin resistance (HOMA-IR; bottom) indices in children as a function of parental weight status at conception. *n* lean–lean = 106; overweight–lean = 184; lean–overweight = 114; overweight–overweight = 415. Data represented as mean ± s.e.m. Significance calculated by two-way ANOVA (details in the graph). Source Data

To study the susceptibility of epididymal spermatozoa to the environment and dissect the relative contribution of epididymal and spermatogenic information to paternal intergenerational effects, we fed 6-week-old male mice with HFD or low-fat diet (LFD) for 2 weeks (Fig. 1a). After the dietary challenge, treated bucks were either directly mated to age-matched and unexposed dams to generate the F_1_ generation (eHFD) or mated (to empty the epididymis) and moved back to a normal chow diet for 4 weeks to allow any HFD-exposed developing germ cell to complete the differentiation and the epididymal maturation before mating (sHFD; Fig. 1a).

**Extended Data Fig. 1:**
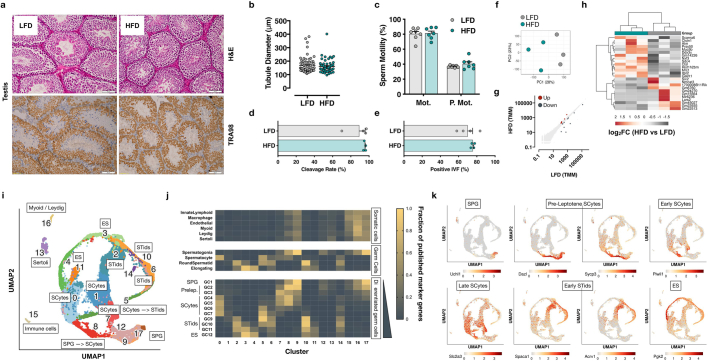
(Extended Data Fig. 1 - related to Fig. 1) - Acute HFD treatment does not affect spermatogenesis. (**a**) Representative H&E (top) and TRA98 (bottom) staining in testes from LFD (left) or HFD (right) fed mice. N = 6-7 mice/group independently stained and analysed (**b**) Quantification of the testis tubule diameter (average of the horizontal and vertical diameters) from H&E-stained testes (N = 60–70 seminiferous tubules – 10/mouse/group – significance calculated with Mann-Whitney test to compare ranks). (**c**) Motility of cauda spermatozoa from LFD and HFD-fed mice (N = 7 – significance calculated by two-tailed t-test). (**d**-**e**) First cleavage (d) and positive IVF (% of fertilised oocytes that complete pre-implantation development - e) rates in embryos generated via IVF with sperm from LFD or HFD-fed mice (N = 4 IVF each done with a pool of spermatozoa from 10 mice – significance calculated by two-tailed t-test). (**f**) PCA representation of the RNA-seq analysis of Round Spermatids (RS) isolated from testes of mice fed with LFD or HFD (f). (**g**-**h**) Scatter plot (g) and heatmap (h) representation of differentially expressed genes in round spermatids. (**i**-**j**) UMAP representation (i) and cluster annotation (j - based on publicly available datasets^64^ - GSE112393) of single-cell RNA-seq analysis of testes from LFD and HFD-fed mice (n = 3). (**k**) Representative marker genes for the different germ cell populations. Source Data

Spermatogenesis and male reproductive fitness are not affected by the HFD-feeding, as shown by the testis histology and the diameter of the seminiferous tubule (Extended Data Fig. 1a,b), sperm motility (Extended Data Fig. 1c) and the rates of successful fertilization and pre-implantation development (Extended Data Fig. 1d,e). In keeping with this, bulk (Extended Data Fig. 1f–h) and single-cell (Extended Data Fig. 1i,k) transcriptomic analyses of round spermatids and testis show normal spermatogenesis (Extended Data Fig. 1f–k) and little to no HFD effect (Supplementary Table 2).

**Extended Data Fig. 2:**
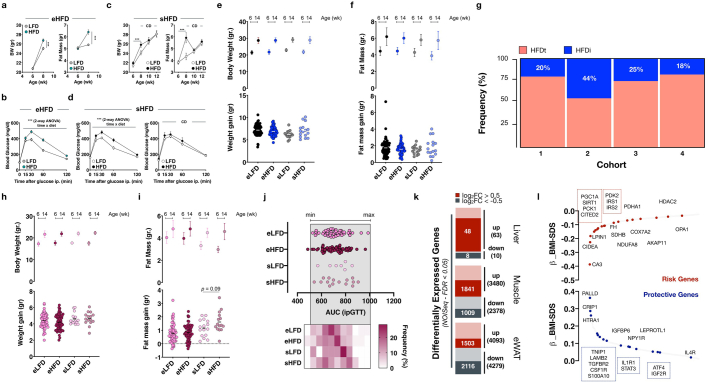
(Extended Data Fig. 2 - related to Fig. 1) - Paternal overweight at conception is important for offspring metabolism in mice. (**a**-**b**) Body weight (a-left), body composition (a-right) and glucose tolerance (b) in mice exposed to two weeks of LFD or HFD feeding. (mean ± SEM – significance calculated by two-tailed t-test (a; N = 18/diet – p-value = 0.0003 BW, and <10^−4^ Fat mass) or two-way ANOVA (b; N = 10-11/diet – p-value < 10^−4^)) (**c**-**d**) Effect of four weeks recovery on chow diet on diet-induced body weight (c-left), body composition (c-right) and glucose tolerance (d). N = 8/diet (mean ± SEM – significance calculated by two-tailed t-test (c – p-value = 0.03 BW, and 0.008 Fat mass) or two-way ANOVA (d – p-value = 0.006)). (**e**-**f**) Body weight (e) and Fat mass (f) development in male offspring of LFD and HFD-exposed bucks and bucks allowed to recover for four weeks on chow diet (N = 60 male mice across 4 cohorts with 5 litters each and 3 males/litter (eLFD and eHFD); N = 10–15 (sLFD and sHFD) including one cohort with 5 litters and 3 males/litter – significance calculated with Mann-Whitney test to compare ranks for weight and fat-mass gain). (**g**) Frequency of HFDi offspring across four different F0-F1 cohorts. (**h**-**j**) Body weight (h), Fat mass (i) and glucose tolerance (j) in female offspring of LFD and HFD-exposed bucks and bucks allowed to recover for four weeks on chow diet. (N = 60 female mice across 4 cohorts with 5 litters each and 3 female/litter (eLFD and eHFD); N = 10–15 (sLFD and sHFD) including one cohort with 5 litters and 2-3 females/litter – significance calculated with Mann-Whitney test to compare ranks for weight and fat-mass gain). (**k**) Differentially Expressed Genes (male HFDi vs HFDt) in peripheral tissues. (**l**) Childhood obesity risk (top) and protective (bottom) genes differentially expressed in HFDi tissues. Source Data

A 2-week exposure to HFD is sufficient to induce a small but significant increase in body weight and adiposity (Extended Data Fig. 2a,c) and reduce whole-body glucose tolerance (Extended Data Fig. 2b,d) in exposed mice. Both phenotypes are reversed by 4 weeks of dietary restoration (Extended Data Fig. 2c,d, chow diet).

An HFD challenge of 2 weeks in bucks (eHFD) does not affect offspring body weight and composition (Extended Data Fig. 2e,f,h,i), but leads to an approximately 30%-penetrant glucose intolerance in male offspring (Fig. 1b and Extended Data Fig. 2j), according to which they are divided into HFD tolerant (HFDt) and HFD intolerant (HFDi; Fig. 1b). The glucose intolerance phenotype is robust as it is observed within litters across four different cohorts in different seasons and different mouse rooms (Extended Data Fig. 2g), and stable as HFDi mice remain significantly glucose intolerant when re-phenotyped 8 weeks after the first phenotyping round (Fig. 1c). HFDi mice are also significantly insulin resistant compared to the other groups (Fig. 1d). Conversely, offspring of sHFD bucks show no alteration in body weight, body composition or glucose tolerance (Fig. 1b and Extended Data Fig. 2e,f,h–j).

The phenotypic differences between HFDt and HFDi offspring are mirrored by unique transcriptional signatures in metabolically relevant tissues (Fig. 1e,f and Extended Data Fig. 2k). Using gene expression and genome-wide association study data from children included in the Leipzig Adipose Tissue Childhood cohort^30^, we found that about 30% of the HFDi signature genes (defined as differentially expressed in both muscle and adipose tissue from HFDi mice) are also expressed in human adipocytes and associated with childhood obesity (Fig. 1g). In this gene set, risk and protective genes for childhood obesity (respectively with negative and positive *β*-scores for BMI standard deviation score (SDS)) cluster to cellular mitochondrial function and inflammatory and cellular plasticity pathways, respectively (Fig. 1g and Extended Data Fig. 2l).

## Paternal BMI affects offspring health

Parental obesity, especially maternal, is the strongest risk factor for early-onset obesity in children^31^. To provide further evidence for paternal contribution to offspring metabolism in humans, we analysed data from the LIFE Child Study (*n* = 3,431; NCT02550236)^32,33^.

**Extended Data Fig. 3:**
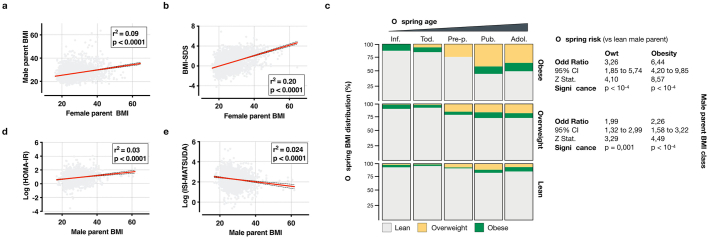
(Extended Data Fig. 3 - related to Fig. 1) - Paternal overweight at conception is important for offspring metabolism in humans. (**a-b**) Control linear regression analysis (r^2^ and *p*-value in the respective panels) for associations between (a) maternal and paternal BMIs at conception and (b) maternal and offspring BMI (dotted lines indicate the 95% confidence intervals of the regression line). (**c**) Frequency of overweight and obesity in offspring stratified by paternal BMI groups in families with lean mothers (analysis detailed in the methods section; results in the figure). (**d**-**e**) Control linear regression analysis (r^2^ and *p*-value in the respective panels) for associations between ISI-MATSUDA (d) and HOMA-IR (e) insulin sensitivity indices and paternal BMI at conception (dotted lines indicate the 95% confidence intervals of the regression line).

Despite the strong association of maternal BMI with both paternal and offspring BMI (Extended Data Fig. 3a,b), the latter is also independently correlated to paternal BMI (Fig. 1h). Multiple regression analysis confirmed that paternal BMI has an additional effect on offspring BMI (6.5%), independent from maternal BMI (20.4%) and age (2.3%) of the offspring (Supplementary Table 6).

In families with lean mothers, paternal overweight doubles offspring obesity risk (Extended Data Fig. 3c; OR = 2.26; *P* value < 10^−4^), which is further worsened by paternal obesity (Extended Data Fig. 3c; OR = 6.44; *P* value < 10^−4^). An increasing paternal BMI is also associated with insulin resistance (as measured by the ISI_Matsuda_ and the HOMA-IR indices)^34^ with parental BMIs having independent and additive effects on offspring insulin sensitivity (Fig. 1i and Extended Data Fig. 3d,e). The effects of both parents remain significant after adjusting for relatedness and polygenic effects (Supplementary Table 7). Mothers explain 18% of BMI-SDS variance and fathers explained 6% of the variance independent from the mothers (both with a *P* value < 0.001).

These results strengthen the importance of paternal preconceptional body weight for offspring metabolic health in mice and humans. Furthermore, the phenotypic discrepancy between offspring sired by mice exposed to 2 weeks of HFD (eHFD) and offspring sired by those then allowed to recover on chow diet for 4 weeks (sHFD) not only shows that this diet-based model is fully reversible but also suggests that epididymal spermatozoa can be directly susceptible to environmental cues.

## mt-tsRNAs are sperm-borne

**Fig. 2:**
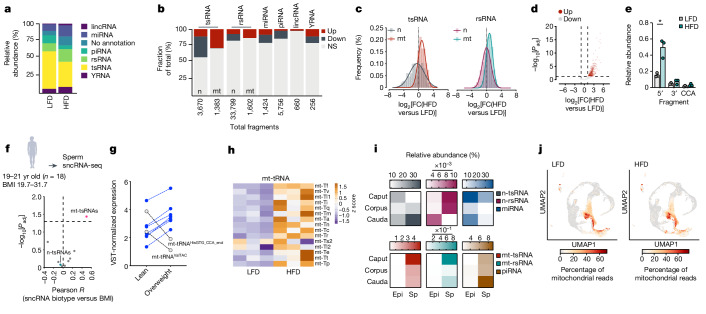
mt-tsRNAs are sperm-borne sensors of male health. **a**, Distribution of sncRNA biotypes in cauda spermatozoa from LFD- and HFD-fed mice. *n* = 3 samples per diet. lincRNA, long intergenic non-coding RNA; miRNA, microRNA. **b**, Biotype-specific differential expression analysis (*P*_adj_ < 0.05; log_2_[fold change (FC)] > |1|) of cauda spermatozoa sncRNAs. **c**, Histogram (bars at a bin centre of 0.5 with the overlapping non-linear fit curve) showing the distribution of the log_2_[FC(HFD versus LFD)] in nuclear- (n) and mitochondrial- (mt) derived tsRNAs (left) and rsRNAs (right). **d**, Volcano plot representation of differentially expressed mt-tsRNAs (differential expression calculated by EdgeR; significance defined as *P*_adj_ < 0.05). **e**, Fragmentation pattern of mt-tRNAs in cauda spermatozoa from LFD- and HFD-fed mice. Significance tested by a two-tailed *t*-test, HFD versus LFD (mean ± s.e.m.; *n* = 3; *P < 0.05). **f**, Pearson-based correlation analysis of individual sncRNA biotype expression in human sperm with BMI (two-tailed *P* value with 95% confidence interval). **g**, Differentially expressed tsRNAs in human sperm from lean and overweight donors calculated by DESeq2-based continuous differential expression analysis (*n* = 18 donors). VST, variance-stabilizing transformation. **h**, Heat map representation of mature mt-tRNA levels in cauda spermatozoa from LFD- and HFD-fed mice. **i**, Relative abundance of the indicated sncRNA biotypes in epididymosomes (Epi) or spermatozoa (Sp) isolated from the caput, corpus and cauda epididymis. Data reanalysed from refs. ^13,38^. **j**, Uniform manifold approximation and projection (UMAP) representation of mtDNA transcription during spermatogenesis from testis single-cell RNA-seq data.

**Extended Data Fig. 4:**
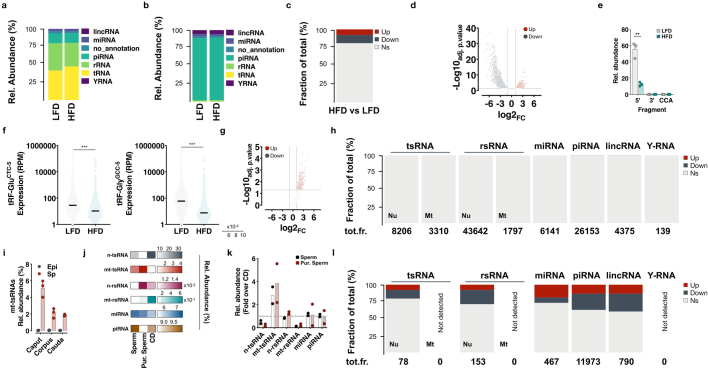
(Extended Data Fig. 4 - related to Fig. 2) - SncRNA-sequencing analysis of mouse cauda spermatozoa and purified round spermatids. (**a**-**b**) Distribution of sncRNA biotypes in cauda spermatozoa from LFD and HFD-fed mice after recovery on chow diet (a - N = 3 samples/diet) and in purified round spermatids from LFD and HFD-fed mice (b - N = 3 samples/diet). (**c**) Differential expression analysis of cauda spermatozoa sncRNA sequencing from LFD and HFD-fed mice. (**d**) Volcano plot representation of DE n-tsRNAs. (**e**) Fragmentation pattern of n-tRNAs in cauda spermatozoa from LFD and HFD-fed mice. Significance tested by a two-tail t-test HFDvsLFD (p-value = 0.0017, N = 3). (**f**) Violin plots illustrating the downregulation of tRF-Glu^CTC-5^ (left) and tRF-Gly^GCC-5^ (right) in spermatozoa from HFD-fed mice. Significance tested by a two-tailed Mann-Whitney test to compare ranks (p-value < 10^−4^). (**g**) Volcano plot representation of DE mt-rsRNAs. (**h**) Biotype-specific differential expression analysis of cauda spermatozoa sncRNAs from LFD and HFD-fed mice after recovery on chow diet. (**i**) Bar plot showing the relative abundance of mt-tsRNAs in epididymosomes or spermatozoa from caput, corpus and cauda epididymis (N = 3)^13,38^. (**j**) Relative abundance of the indicated sncRNA biotypes in cauda spermatozoa, its associated cytoplasmic droplets (CD) and CD-depleted cauda spermatozoa (pur. Sperm). Data re-analysed from Wang H. et al. ^16^. (**k**) Enrichment of the indicated small RNAs in cauda spermatozoa over its associated cytoplasmic droplets (N = 2)^16^. (**l**) Biotype-specific differential expression analysis of round spermatids sncRNAs from LFD and HFD-fed mice.

sncRNAs in spermatozoa are potential diet-sensitive mediators of paternal epigenetic effects^1,35^. We profiled sncRNAs from round spermatids and cauda spermatozoa of mice fed for 2 weeks on HFD (eHFD-F_0_), and from cauda spermatozoa of mice fed for 2 weeks on HFD and allowed to recover on chow diet for 4 weeks (sHFD-F_0_). The biotype distributions in the different samples followed the expected profiles (Fig. 2a and Extended Data Fig. 4a,b) with tsRNAs and piwi-RNAs (piRNAs) being the most abundant biotypes in spermatozoa (Fig. 2a and Extended Data Fig. 4a) and round spermatids (Extended Data Fig. 4b), respectively. Differential expression analysis of cauda spermatozoa sncRNA-sequencing (sncRNA-seq) data from eHFD-F_0_ mice indicates that about 25% of the entire sperm sncRNA pool is sensitive to the acute HFD challenge (Extended Data Fig. 4c) with predominant reduction of nuclear tRNA expression (Fig. 2b,c and Extended Data Fig. 4d) and fragmentation (Extended Data Fig. 4e), including 5′ fragments previously associated with the intergenerational effects of paternal overt obesity^5^ (for example, GluCTC and GlyGCC; Extended Data Fig. 4f). Conversely, expression and 5′ fragmentation of mt-tRNAs are predominantly upregulated (Fig. 2c–e) with about 30% of annotated sequences reaching statistical significance (false discovery rate < 0.05; Fig. 2b,d). In line with these data, our results show that fragments derived from mitochondrial rRNAs are also upregulated (Fig. 2c and Extended Data Fig. 4g) with about 20% of annotated sequences reaching statistical significance (false discovery rate < 0.05; Fig. 2b). Differential expression analysis of cauda spermatozoa sncRNA-seq from HFD-fed mice after recovery on chow diet for 4 weeks (sHFD-F_0_) shows little to no variation for any of the biotypes (Extended Data Fig. 4h).

In conjunction with the offspring phenotypes (Fig. 1 and Extended Data Fig. 2), these findings support a primary role of the epididymis in the response to the acute HFD challenge and suggest a role for sperm sncRNAs as dynamic molecular signals.

**Extended Data Fig. 5:**
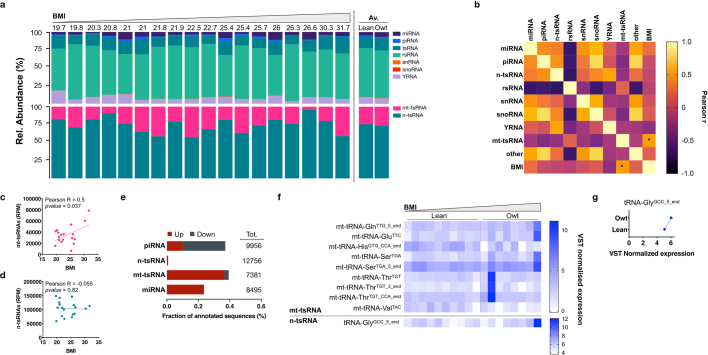
(Extended Data Fig. 5 - related to Fig. 2) - SncRNA-sequencing analysis of human spermatozoa. (**a**) Distribution of sncRNA biotypes in human spermatozoa purified from ejaculates. (up, left) biotypes distribution in single donors stratified per BMI; (up, right) average biotypes distribution in lean (BMI < 24) and owt (BMI > 24) donors. (down, left) relative abundance of n- and mt-tsRNAs in single donors stratified per BMI; (down, right) average relative abundance of n- and mt-tsRNAs in lean and owt donors. (**b**-**d**) *Pearson*-based co-correlation matrix of sncRNA biotypes and BMI (b) and scatter plot representation for mt-tsRNAs (c) and n-tsRNAs (d) Exact p-values for c and d indicated in the figure. (**e**) Biotype-specific differential expression analysis of human sperm sncRNAs. (**f**) Heatmap representation of the Continuous Differential Expression analysis results for n- and mt-tsRNAs. (**g**) Dot plot showing the upregulation of n-tRNA-Gly^GCC_5_end^ in spermatozoa from owt donors.

In *Drosophila melanogaster* and human spermatozoa, mt-tsRNAs are dynamically upregulated by a short high-sugar diet^35,36^. To further study their association with BMI, we profiled ejaculate spermatozoa sncRNAs from young, healthy Finnish volunteers (*n* = 18, age range 19–21 years) deeply metabolically phenotyped and stratified per BMI and fat mass (Supplementary Table 4). Relative biotype distribution and tsRNA pool composition are comparable across individuals and BMI categories (Extended Data Fig. 5a). As BMI is a continuous variable, we used continuous differential expression analysis algorithms to identify sncRNAs in significant linear association with BMI variation. Together with little to no variation in n-tsRNAs (Extended Data Fig. 5e–g), mt-tsRNAs are the only biotype positively associated with BMI (Fig. 2f and Extended Data Fig. 5b–d). A total of 0.5% of sequences annotated to mt-tsRNAs are differentially expressed with continuous BMI variation (Extended Data Fig. 5e), accounting for a total of 29 individual sequences clustered to 9 fragments, of which 7 are upregulated (Fig. 2g and Extended Data Fig. 5f). Of note, the downregulated sequences (Fig. 2g) are also the only ones downregulated in mouse spermatozoa following 2 weeks of HFD-feeding, whereas among the upregulated sequences, mt-tRNA^Ser^- and mt-tRNA^Thr^-derived fragments are also upregulated by an acute high-sugar challenge^35^ and downregulated in spermatozoa from overly obese individuals^37^.

Although obtained from a small human cohort, these findings reinforce the idea that mt-sncRNAs can have important functions in response to acute metabolic challenges in mice and humans.

Nuclear-encoded tRNAs are mainly acquired by spermatozoa from epididymosomes or cytoplasmic droplets during the epididymal transit^4,10,11,13–16^. Conversely, mt-tRNAs can be actively transcribed in spermatozoa, as mtDNA transcription is specifically active in mature spermatozoa^27^. Indeed, full-length mt-tRNA transcripts are detected by standard RNA-seq in mature spermatozoa and upregulated following 2 weeks of HFD-feeding (Fig. 2h). Furthermore, reanalysing publicly available sncRNA-seq datasets generated from parallel epididymal sampling of epididymosomes and spermatozoa^13,38^ or sncRNA-seq of cauda spermatozoa and their associated cytoplasmic droplets^16^ we found mt-tsRNAs and mt-rsRNAs, almost exclusively in spermatozoa (Fig. 2i and Extended Data Fig. 4i–k). This is in keeping with sncRNAs of germline origin, such as piRNAs (Fig. 2i) and in contrast to sncRNAs of somatic or mixed origin, such as microRNAs and n-tsRNAs (Fig. 2i). Confirming previously published findings^39^, our testis single-cell RNA-seq data also show that mtDNA transcription stops at the spermatocyte stage (Fig. 2j; see Extended Data Fig. 1i–k for spermatocyte annotation), without differences following HFD (Fig. 2j). Together with no detection of mt-sncRNAs in round spermatids (Extended Data Fig. 4l), these findings are against a possible flux of mt-sncRNAs from spermatogenesis to mature spermatozoa. They instead support a primary function of mature spermatozoa in sensing the environment—most likely through diet-induced modifications of the epididymal microenvironment^40^—and add sperm-borne mt-sncRNAs to the pool of potential transducing factors across generations.

## Epigenetic inheritance of mt-tRNAs

**Fig. 3:**
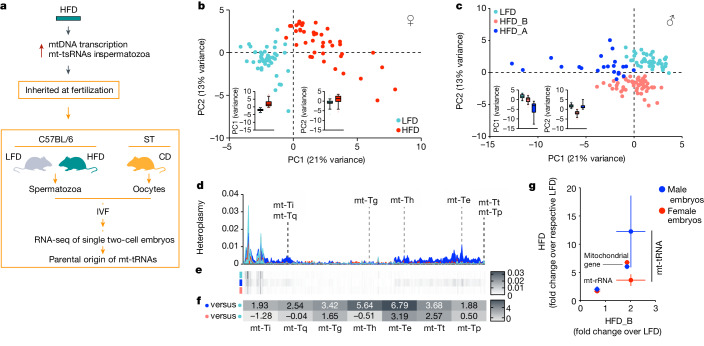
Sperm mt-tRNA sequences are transferred to the oocytes at fertilization. **a**, Experimental design to generate and analyse single hybrid early two-cell embryos generated through IVF with BL6 sperm (from LFD- or HFD-fed mice) and ST oocytes. **b**,**c**, PCA plot representation of mitochondrial transcriptomes in single female (**b**) and male (**c**) embryos. Insets represent the variance at the two main principal components (whiskers are the 5th and 95th percentile of the distribution; experimental and analytical details in the Methods). **d**,**e**, Density plot (**d**) and heat map (**e**) representation of the quantified heteroplasmy at the 416 SNPs mapped between BL6 and ST mitochondrial genomes. **f**, Relative heteroplasmy enrichment of the highlighted mt-tRNAs against LFD embryos. **g**, Biotype-specific heteroplasmy enrichment (over LFD) in male and female HFD embryos (HFD_B shown on the *x* axis; data represented as mean ± s.e.m.).

To study the intergenerational transfer of sperm-borne mtRNAs, we harnessed the genetic diversity of individual mtDNAs, and the maternal inheritance of mtDNA^41^. We generated hybrid embryos through in vitro fertilization (IVF) using cauda spermatozoa from eHFD-F_0_ and LFD-F_0_ C57BL/6N bucks and oocytes from inbred, homoplastic Staudach (ST; C57BL/6N-mt^ST^) dams. The ST and BL6 mtDNAs differ by 416 single nucleotide polymorphisms (SNPs)^42^ distributed along the mtDNA and present in some of the mt-tRNAs differentially expressed in cauda spermatozoa from eHFD-F_0_ mice. We then profiled the transcriptomes of about 200 single hybrid early two-cell embryos (embryonic day 1.5), to track the parental origin(s) of mtRNAs using the SNPs differentiating the two parental mouse strains (Fig. 3a).

**Extended Data Fig. 6:**
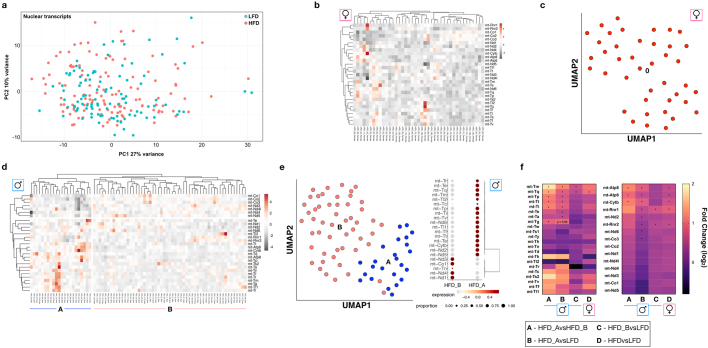
(Extended Data Fig. 6 - related to Fig. 3) - Male embryos sired by HFD-fed bucks cluster into two independent clusters characterized by differential expression of mt-tRNAs. (**a**) PCA plot representation of nuclear transcriptomes in single male and female hybrid embryos. (**b**-**c**) Hierarchical (b) and Seurat-based (c) clustering of female embryos sired by HFD-fed fathers. Both methods identify one single cluster in female embryos. (**d**-**e**) Hierarchical (d) and Seurat-based (e) clustering of male embryos sired by HFD-fed fathers. Both methods identify two independent clusters in male embryos defined by differential expression of mt-tRNAs. (**f**) Heatmap representation of mt-tRNAs (left) and mt-genes (right) expression in the different embryo populations. Differential expression calculated with the DESeq2 algorithm. * = adj.*p value* < 0.05 (see methods for details).

**Extended Data Fig. 7:**
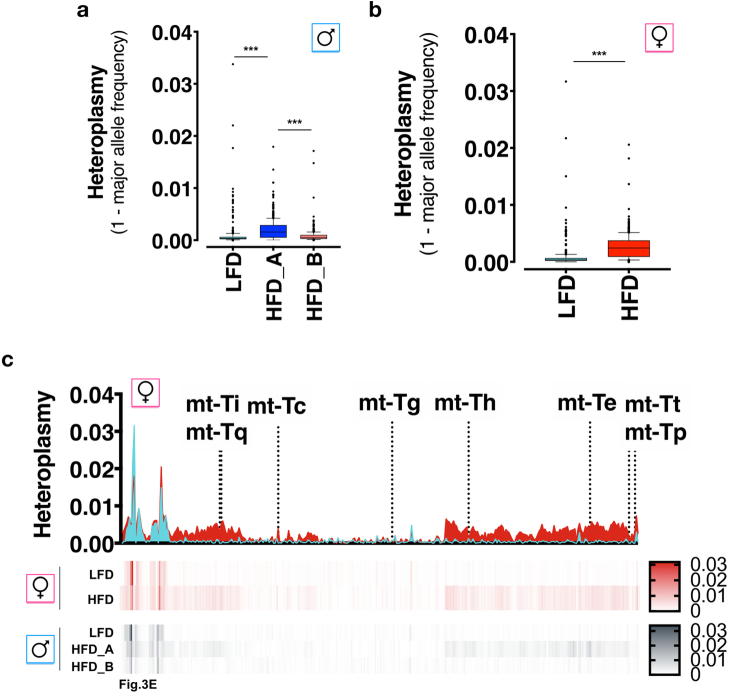
(Extended Data Fig. 7 - related to Fig. 3) - Sperm mt-RNAs are transferred to the oocytes at fertilization. (**a**-**b**) Average heteroplasmy in male (a) and female (b) early 2CE. (Whiskers are the 10^th^–90^th^ percentile of the data distribution. N = 373 SNPs with quantifiable heteroplasmy. Significance calculated by two-tailed paired t-test. **** = *p value* < 10^−4^). (**c**) Density plot (top) and heatmap (bottom) representation of the quantified heteroplasmy in female HFD and LFD embryos at the 416 SNPs mapped between BL6 and ST mitochondrial genomes. mt-tRNAs are highlighted. For comparison between male and female embryos, the heatmap from Fig. 3e is also shown.

Principal component analysis (PCA)-based clustering of the single embryos using the nuclear transcriptomes shows complete overlap between embryos sired by LFD- or HFD-fed bucks (Extended Data Fig. 6a), suggesting no global paternal effect. The same analysis using mitochondrial transcriptomes highlights differential clustering between LFD- and HFD-sired female and male embryos (Fig. 3b,c). Whereas HFD-sired female embryos are homogeneously grouped into one single cluster (Fig. 3b and Extended Data Fig. 6b,c), male embryos form two independent clusters (Fig. 3c and Extended Data Fig. 6d,e), with HFD_A embryos (dark blue, about 30% of the HFD male embryo population) being characterized by a significant overexpression of mt-tRNAs (Extended Data Fig. 6d–f). To study the paternal contribution to the embryonic pool of mtRNAs, we quantified the frequency of paternal alleles (expressed as heteroplasmy = 1 − maternal allele frequency) at the 416 SNPs in the different embryo populations. Male HFD_A embryos show increased heteroplasmy compared to both HFD_B and LFD embryos (Extended Data Fig. 7a) especially at SNPs falling into mt-tRNAs (Fig. 3d–g). Female HFD embryos also show increased heteroplasmy compared to LFD embryos (Extended Data Fig. 7b,c), while featuring reduced heteroplasmy at mt-tRNAs (Fig. 3g) when compared to HFD_A embryos (HFD_A = 12×, HFD_B = 2×, HFD_female = 3.5× compared to the respective LFD).

With the caveat that other sperm RNAs can also be paternally inherited, these findings demonstrate—in a physiological setting—sperm-to-oocyte transfer of mtRNAs at fertilization.

The male-specific and 30%-penetrant offspring phenotype makes the preferential transfer of sperm mt-tRNAs to the HFD_A subpopulation of male embryos (Fig. 3g) intriguing.

## Analysis of early-embryo transcription

**Fig. 4:**
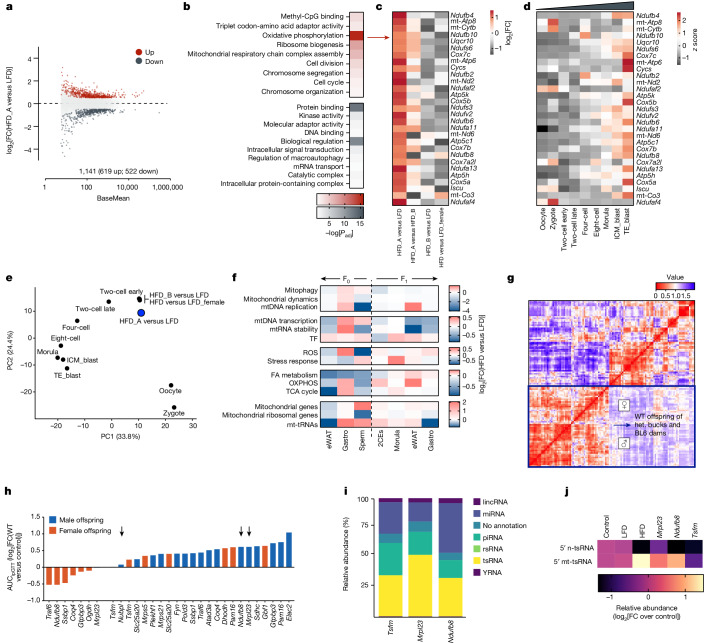
Double-edge connection between mitochondrial metabolism and paternal epigenetic inheritance. **a**, MA plot representation of differentially expressed genes in HFD_A versus LFD embryos. **b**, Gene Ontology-based functional enrichment analysis of differentially expressed genes in HFD_A versus LFD embryos (see Methods for details). **c**,**d**, Heat map representation of the relative expression (HFD versus LFD) of OXPHOS genes in early two-cell embryos (**c**) and in a publicly available dataset^58^ of single-embryo RNA-seq analysis of mouse pre-implantation development (**d**). TE_blast, trophectoderm blastocyst; ICM_blast, inner cell mass blastocyst. **e**, PCA-based representation of the developmental timing in HFD and LFD embryos laid over the analysis of mouse pre-implantation development of ref. ^58^. **f**, RNA-seq-based quantification of the expression of genes important for mitochondrial function across adult tissues and germ cells of male mice exposed to 2 weeks of HFD as well as early embryos and adult tissues from unexposed male offspring of HFD-fed fathers. 2CEs, two-cell embryos; TF, transcription factor; ROS, reactive oxygen species; gastro, gastrocnemius. **g**, Pearson-based correlation matrix of IMPC-derived metabolic phenotypes in WT offspring of parents heterozygous (het.) for IMPC-selected genes (see Methods for details). **h**, Bar plot representation of the relative (WT versus control) glucose intolerance (measured as AUC_ipGTT_) in WT offspring of fathers heterozygous for genes important for mitochondrial structure and function. Black arrows indicate genes for which cryopreserved heterozygous sperm samples were analysed. **i**, Distribution of sncRNA biotypes in cauda spermatozoa from the indicated mutant mice (*n* = 10 mice per gene). **j**, Heat map representation of the relative abundance of 5′ n-tsRNAs and 5′ mt-tsRNAs in mutant spermatozoa. Control, LFD and HFD samples are cryopreserved and resequenced to serve as reference and technical controls.

**Extended Data Fig. 8:**
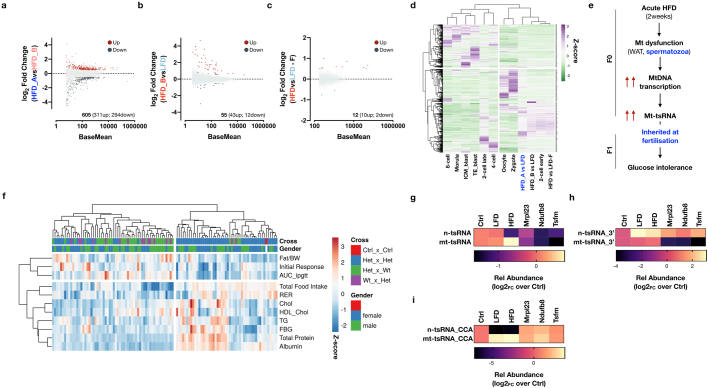
(Extended Data Fig. 8 - related to Fig. 4) - Double-edge connection between mitochondrial metabolism and paternal epigenetic inheritance. (**a**-**c**) MA plot representation of differentially expressed genes in HFD_A vs HFD_B (a), HFD_B vs LFD (b) and HFD vs LFD Female (c) embryos. (**d**) Heatmap representation of the genes used for the PCA plot in Fig. 4e. (**e**) Scheme of the working hypothesis by which exposure of male mice to two weeks of HFD induces mild mitochondrial dysfunction, which leads to a compensatory up-regulation of mt-DNA transcription in spermatozoa, transfer of mt-tRNAs from sperm-to-oocytes at fertilisation and reprogramming of offspring glucose metabolism. (**f**) Heatmap representation of the IMPC metabolic phenotypes analysed in the WT offspring of IMPC-selected genes mutants with indication of offspring gender and parental effects (**Het_x_Wt = paternal effect**). (**g**-**i**) Heatmap representation of the tsRNA expression (g) and the relative abundance of 3’ (h) and CCA (i) fragments in mutant spermatozoa.

Differential gene expression analysis revealed substantial transcriptional reprogramming in HFD_A embryos compared to both LFD (Fig. 4a) and HFD_B (Extended Data Fig. 8a), with little to no variation in both HFD_B or female embryos when compared to their respective LFD controls (Extended Data Fig. 8b,c). Gene Ontology analysis of differentially expressed genes highlighted clustering of genes upregulated in HFD_A embryos to cellular metabolism terms (Fig. 4b), with oxidative phosphorylation (mostly Complex I; GO:0006119) being the top one (adjusted *P* value (*P*_adj_) < 10^−16^; Fig. 4b,c). Mammalian pre-implantation development represents an example of continuous metabolic adaptation^43^ in which developing embryos face a gradient of oxygen concentration from the oviduct to the uterus resulting in a sixfold increase in oxidative phosphorylation in blastocysts compared to cleavage-stage embryos^44^ (Fig. 4d). Premature activation of oxidative phosphorylation during pre-implantation development leads to alterations in embryo ultrastructures^45^, mitochondrial structure and function^46^ and glucose intolerance in adult mice^47^; and paternal diet can influence pre-implantation developmental timing and early-embryo metabolism^48^. Although the developmental timing of HFD_A embryos is maintained (Fig. 4e and Extended Data Fig. 8d), their gene expression profile suggests premature activation of oxidative metabolism associated with paternal overweight and inheritance of mt-tRNAs (Fig. 3g).

## Mitochondrial dysfunction mimics HFD

Oxidative phosphorylation is essential to cellular and organismal physiology^49^. Acute and chronic exposure of mice to HFD affect metabolic homeostasis and mitochondrial function in the brain^50^, peripheral tissues^51^ and germ cells^52^. Cells also try to compensate mitochondrial dysfunction by upregulating transcription of mtDNA^53^, as occurs in mature spermatozoa, which need healthy mitochondria for proper fertilization capacity^54^.

Comparing tissue transcriptomics from treated and control animals across two generations, we found consistent downregulation of genes important for mitochondrial metabolism in diet-exposed mice (for example, fatty acid metabolism; Fig. 4f). In muscle and cauda spermatozoa, this is coupled to upregulation of the mtDNA transcriptional machinery (Fig. 4f). Early embryos and F_1_ somatic tissues instead show consistent upregulation of genes important for mitochondrial metabolism (Fig. 4f right side), whereas only early embryos maintain the paternal upregulation of mt-tRNAs (Fig. 4f).

From these results, we reason that the upregulation of mt-tRNAs (and their 5′ fragments) in cauda spermatozoa is a compensatory response to the diet-induced mitochondrial dysfunction (Extended Data Fig. 8e).

To test the hypothesis that paternal mitochondrial dysfunction could affect sperm mt-tRNAs and induce intergenerational glucose intolerance, we used the systemic phenotype data resource of the IMPC^29^ and extracted metabolic parameters measured in wild-type (WT) offspring of parents heterozygous for genes involved in mitochondrial structure and function. With most of these genes being lethal in homozygosity^55^ and/or compromising fertility^55^, our final dataset included parent-of-origin information. Pearson-based co-correlation analysis of WT offspring phenotypes indicated specific paternal effects (Fig. 4g, bottom left cluster) with increased adiposity (fat/body weight) and glucose intolerance (area under the curve of an intraperitoneal glucose tolerance test (AUC_ipGTT_)) mainly in male offspring (Fig. 4h and Extended Data Fig. 8f). The mutant lines phenotyped by the IMPC are archived as cryopreserved sperm and available to the community through the European Mouse Mutant Archive (EMMA^56^). We obtained frozen sperm for three lines: *Mrpl23* (mitochondrial ribosomal protein L23), *Ndufb8* (NADH:ubiquinone oxidoreductase subunit B8) and *Tsfm* (Ts translation elongation factor, mitochondrial). Whereas paternal manipulation of *Mrpl23* and *Ndufb8* reprograms offspring metabolism, *Tsfm* fails in this process (Fig. 4h), thus serving as a suitable negative control for initial mechanistic dissections. sncRNA-seq of cryopreserved sperm samples from the three mutant lines revealed the expected biotypes distribution (Fig. 4i) and a striking accumulation of 5′ mt-tsRNAs in *Mrpl23* and *Ndufb8* mutants, but not *Tsfm* (Fig. 4j and Extended Data Fig. 8g–i). These results are in keeping with the failure of *Tsfm*-mutant bucks to reprogram offspring glucose metabolism (Fig. 4i) and phenocopy paternal HFD exposure, supporting the proposal that mitochondrial dysfunction triggers the observed intergenerational paternal effects.

## Conclusions and perspectives

This study shows that acute HFD-feeding or genetic induction of mitochondrial dysfunction in male mice leads to impaired glucose homeostasis in male (WT and unexposed) offspring (Figs. 1 and 4). Mechanistically, this is associated with an accumulation of mt-tRNAs transcribed and fragmented in mature spermatozoa (Figs. 2 and 4) and transferred to the oocyte at fertilization (Fig. 3). The resulting early two-cell embryos show significantly altered transcription of genes important for oxidative metabolism (Fig. 4), shown to predispose to adult-onset glucose intolerance^46^.

Evolutionarily, these findings present a fully reversible mechanism (Fig. 1 and Extended Data Figs. 2 and 4) by which male parents update the offspring on their metabolic health by transferring mitochondrial signals and thereby overcoming the mechanisms by which fertilized oocytes eliminate sperm mitochondria^57^. Despite the lack of zygotic microinjections data demonstrating that mt-tsRNAs are sufficient to transfer the metabolic phenotypes, and therefore with the standing caveat that other sncRNAs (and epigenetic factors) can contribute to the observed paternal effects; the robustness, the strength, the dynamic and the reversibility of the mt-tRNA signature makes these sperm-borne RNAs suitable candidates for new strategies to monitor preconception lifestyle interventions aimed at preventing the spread of metabolic disorders through paternal epigenetic inheritance.

## Methods

### HFD–LFD experiment in mice (F_0_–F_1_)

#### Animal housing, diet composition and breeding strategies

C57BL/6N male and female mice were purchased from Charles River Laboratories Germany. All animals were fed ad libitum and housed at constant temperature (22 ± 1 °C) and controlled humidity in ventilated cages on a 12 h:12 h light/dark cycle.

For eHFD treatment, 6-week-old male mice were randomly assigned to two groups fed for 2 weeks with purified HFD (rodent diet with 60 kcal% from fat; Research Diet D12492i) or LFD control diet (rodent diet with 10 kcal% from fat; Research Diet D12450B) and subsequently mated with a single unexposed, virgin female of the same age. For sHFD treatment, 6-week-old male mice were randomly assigned to two groups fed for 2 weeks with purified HFD (rodent diet with 60 kcal% from fat; Research Diet D12492i) or LFD control diet (rodent diet with 10 kcal% from fat; Research Diet D12450B), mated with a single unexposed virgin female (to empty the epididymis) and moved back to a standard chow diet for 4 weeks. These animals were subsequently mated with a single, virgin and aged-matched female to generate the offspring cohort.

At the time of mating, both males and females were fed ad libitum on a standard chow diet. To avoid isolation during gestation, males were removed from the cage after delivery and the mothers were maintained individually throughout newborn nursing and lactation. Litter size was adjusted to 6–8 whenever the number of pups was higher, to avoid undernourishment.

Offspring from LFD or HFD males and WT unexposed females were named F_1_. F_1_ animals were weaned at 21 days post-partum and kept on ad libitum chow diet for their entire life.

All animal experiments have been carried out according to the European Union directive 2010/63/EU and were approved by the responsible authorities of the government of Upper Bavaria, under licence number ROB-55.2-2532.Vet_02-17-33.

All efforts have been made to minimize suffering by considerate housing and husbandry. All phenotyping procedures were examined for potential refinements. Animal welfare was assessed routinely for all mice involved. Data from animal experiments are reported in accordance with the ARRIVE guidelines^59^.

#### Phenotyping pipeline

Body weight and relative lean and fat mass were measured in exposed F_0_ animals before and after the dietary challenge, as well as in chow-fed F_1_ animals bi-weekly from 4 to 14 weeks of age. Body composition was determined by nuclear magnetic resonance spectroscopy with a Minispec NMR analyser (Brucker Optics), according to the manufacturer’s instructions. ipGTTs were conducted on 8- or 12-week-old F_0_ mice and 16- and 24-week-old F_1_ mice after an overnight fasting period of 16 h (from 6 pm to 10 am). A ratio of 2 g of glucose per kilogram of fasting body weight was injected. Blood glucose levels were determined before and after the injection at 15, 30, 60 and 120 min using the Roche AccuChek Aviva blood glucose meter. Plasma samples were separated from the whole blood, collected in EDTA-coated microvettes (Sarstedt) at time 0, 30 and 60 min, and snap-frozen in liquid nitrogen for further analyses.

Insulin tolerance tests were carried out on 25-week-old F_1_ mice after 6 h fasting (from 6 am to 12 pm). Mice were injected with 0.5 U insulin per kilogram of body weight. Blood glucose was measured before the intraperitoneal injection and at 15, 30, 60 and 120 min using the Roche AccuChek Aviva blood glucose meter.

#### Analysis of spermatogenesis and sperm function

Testis from 8-week-old mice exposed to 2 weeks of LFD- or HFD-feeding were dissected and processed for histology (*n* = 5 mice per diet), and purification of round spermatids for RNA and sncRNA-seq (*n* = 3 mice per diet) or further processed for single-cell RNA-seq (*n* = 3 mice per diet).

#### Testis staining and histology

Testis sections were fixed for 48 h in 10% formalin, dehydrated through ethanol series, cleared in xylene and embedded in paraffin. After rehydration, 4-μm sections were stained with haematoxylin and eosin, according to the manufacturer’s instructions. For immunohistochemical analysis, 1.5-μm sections were dewaxed by standard techniques. Heat treatment was carried out for antigen retrieval in sodium citrate buffer. Endogenous peroxidase activity was quenched with 3% H_2_O_2_ in methanol at room temperature for 5 min. Incubation with primary antibodies was carried out overnight at 4 °C in blocking buffer (TBS–Tween 1%), and chromogenic reactions were carried out. Staining was carried out using the automatic Discovery XT (Ventana Systems) stainer, following preset protocols. Sections were subjected to EDTA-based antigen retrieval for 20 min and protein block (Dako, DS9390) for 12 min. Sections were then examined under an Olympus microscope. The primary antibody was TRA98 (Abcam Ab82527; 1:1,000); secondary antibody was rabbit anti-rat IgG H&L (HRP; Abcam Ab6734; 1:1,000). The diameters of 60–70 seminiferous tubuli were measured from cross-sectional areas of TRA98-stained testes and expressed as the average of the horizontal and vertical diameters.

#### Round spermatid isolation

Round spermatids were isolated using a modified density gradient protocol optimized for round spermatids as previously described^60^. A minimum of 85–90% purity was microscopically checked. Total RNA was prepared using the RNAeasy Mini Kit (QIAGEN) according to the manufacturer’s instructions. RNA concentration and integrity were controlled on a Bioanalyzer system (Agilent) and only RNA samples with a RIN (RNA integrity number) value > 7 were considered for downstream applications. A 50 ng quantity of total RNA was used to prepare total (Ribo-minus) and sncRNA-seq libraries as described below.

#### Cauda sperm isolation and analysis

Mature cauda spermatozoa were purified by a swim-up procedure^61^ from mice fed on HFD or LFD for 2 weeks (eHFD) and/or allowed to recover on chow diet for 4 weeks after the dietary challenge (sHFD).

Briefly, the cauda and the vas deferens were cut into small pieces, placed in 500 μl Donners medium (25 mM NaHCO_3_, 20 mg ml^−1^ BSA, 1 mM sodium pyruvate and 0.53% (vol/vol) sodium dl-lactate in Donners stock; Donners stock: 135 mM NaCl, 5 mM KCl, 1 mM MgSO_4_, 2 mM CaCl_2_ and 30 mM HEPES, pH 7.4, filtered through a 0.22-μM filter and stored at room temperature), filled up to 2 ml, centrifuged for 2 min at 1,200 r.p.m. and then incubated at 37 °C for 1 h. Afterwards, 1.5 ml of supernatant (composed mainly of motile sperm) was transferred into a new 1.5-ml tube, centrifuged for 2 min at 1,200 r.p.m. and incubated for 30 min at 37 °C. A 1 ml volume of supernatant was then collected, and centrifuged for 5 min at 4,800 r.p.m. The supernatant was discarded and the pellet was resuspended in a cell lysis buffer (SDS 0.01%, Triton X-100 0.005%, dissolved in RNAse-free water) and incubated on ice for 30 min. Samples were then centrifuged at 4,800 r.p.m., washed with cold 1× PBS, resuspended in 500 μl of TRIzol reagent (Thermo Fisher) and stored at −80 °C until further processing. Total RNA was prepared using the RNAeasy Mini Kit (QIAGEN 74104) or the miRNeasy Micro Kit (QIAGEN 1071023) according to the manufacturer’s instructions.

A 10–15-μl aliquot of PBS-washed motile spermatozoa was used to microscopically check somatic cell contamination (only samples with no visible somatic cells were considered for downstream applications) and for functional analyses. Sperm concentration, motility and progressive motility have been calculated using an automated computer-assisted semen analysis (Hamilton Thorn IVOS II) according to the manufacturer’s instructions.

#### IVF and embryo analysis

Oocyte isolation and IVF were conducted following standardized procedures of the INFRAFRONTIER consortium as previously described^21^. Briefly, male gamete donors were euthanized at 8 weeks of age, after the 2-week exposure to LFD or HFD. Mature sperm cells were obtained from cauda epididymis as described above. WT unexposed female oocyte donors were euthanized the same day at 10–11 weeks of age, after superovulation induced with 7.5 U of pregnant mare serum gonadotropin and 7.5 U of human chorionic gonadotropin before being killed for oocyte collection. The sperm and the oocytes were co-cultured for 4–6 h. Subsequently, the oocytes were transferred and incubated in high-calcium human tubal fluid (HTF) culture medium at 37 °C and 5% CO_2_. First-cleavage (zygote to two-cell embryo) rate and rate of blastocyst development were measured to assess embryonic development and complement sperm functional analysis in determining the effects of HFD on male reproductive fitness.

Morulae were microscopically checked and individually picked after incubating fertilized oocytes in high-calcium HTF for 72 h.

#### Testes single-cell RNA-seq library preparation

Testes from 8-week-old mice fed on HFD or LFD for 2 weeks (*n* = 3 per group) were processed to obtain a single-cell suspension^60^. Briefly, testes were decapsulated and incubated with 1 mg ml^−1^ collagenase IV (3 min, 37 °C), washed twice with warm 1× KREBS (10× KREBS: 3.26 g KH_2_PO_4_, 139.5 g NaCl, 5.89 g MgSO_4_⋅7H_2_O, 50 g dextrose, 3.78 g CaCl_2_⋅2H_2_O, 7.12 g KCl in 2 l water and filtered with 0.22 µm) by sedimentation, incubated with DNAse I plus 0.25% trypsin (Gibco; 15–20 min at 34 °C), filtered with a 40-μm filter, centrifuged at 600*g*, 5 min at 4 °C, washed twice with cold 1× KREBS and resuspended with 1 ml 1× PBS + 0.04% BSA. Ten thousand cells were targeted using the Chromium Next GEM Chip G Single Cell and the Chromium Next GEM Single Cell 3′ Gel Bead Kit v3.1. Chromium Next GEM Single Cell 3′ GEM Kit v3.1 was used for cDNA synthesis and Chromium Next GEM Single Cell 3′ Library Kit v3.1 was used for library preparation following the ChromiumNextGEMSingleCell3_v3.1_Rev_D user guide (10x Genomics). Libraries were verified using a 2100 Bioanalyzer (Agilent). Samples were paired-end sequenced on an Illumina NovaSeq 6000 platform following the number of cycles recommended by 10x Genomics.

#### Testes single-cell RNA-seq analysis

Raw sequencing data were demultiplexed using the Cell Ranger (10x Genomics) mkfastq to obtain fastq files for each sample. Demultiplexed reads were processed and mapped to the mouse genome (refdata-gex-mm10-2020-A), and filtering and unique molecular identifier counting were carried out to obtain gene transcript counts per cell (gene barcode matrix) using the Cell Ranger (version 6.0.1) count function (Supplementary Table 1).

Cell Ranger-filtered count matrices for each sample were imported into R and Seurat R objects (version 4.3.0) were created^62^. For each sample, further filtering was conducted to select high-quality cells. We filtered raw count matrices by excluding cells expressing fewer than 200 detectably expressed genes and genes expressed in fewer than three cells. Cells with more than 40,000 detected features (nFeature_RNA) per cell were excluded. All of the samples were combined using the merge function. Gene expression counts were normalized with a scale factor of 10,000 and a log(1 + *n*) transformation, using the Seurat NormalizeData function. The CellCycleScoring function was used to infer cell cycle phase, as it determines relative expression of a large set of G2/M- and S-phase genes. The highly variable genes were identified using the FindVariableFeatures function. The Seurat object was subsequently scaled and analysed by PCA. After PCA, we used the RunHarmony function in the Harmony R package^63^ for integration.

The top 30 principal components were used to carry out the uniform manifold approximation and projection dimensional reduction. We then constructed the nearest-neighbour graph with the FindNeighbors function with the reduction as ‘harmony’, and dimensionality reduction as 1:30. Clusters were then identified using the FindClusters function with the resolution parameter of 0.3, which resulted in 18 clusters. Markers of each cluster were identified using the FindAllMarkers function with a Wilcoxon rank sum test. Cell types were assigned on the basis of publicly available testes single-cell datasets from age-matched mice^64^. The raw count gene expression data were used to carry out differential gene expression for all of the clusters and bulk using the DESeq2 package (Supplementary Table 2).

#### sncRNA-seq library preparation

A 10–50 ng quantity of total RNA from round spermatids (50 ng; *n* = 3 per group) and cauda spermatozoa (10 ng; *n* = 3 per group) from mice fed on HFD or LFD for 2 weeks or cauda spermatozoa from mice fed on HFD or LFD for 2 weeks, mated and allowed to recover on chow diet for 4 weeks (10 ng; *n* = 3 per group) was used for sncRNA library preparation. Libraries were prepared using the NEBNext Multiplex Small RNA Library Prep Set for Illumina (NEB E7560S) with 5′ and 3′ adaptors diluted 1:5 and 15 PCR amplification cycles. Libraries were verified using a 2100 Bioanalyzer (Agilent) and paired-end (read length =  150 base pairs (bp)) sequenced with the Illumina NovaSeq 6000 platform. Although allowing robust detection of all sncRNA biotypes, this library preparation method does not efficiently capture highly modified sncRNAs, such as tsRNAs and rsRNAs.

#### sncRNA-seq analysis

Raw sequencing data were quality checked using MultiQC v1.11. Reads were trimmed using cutadapt 2.8 according to the kit manufacturer’s instructions. Trimmed and quality-filtered reads were used for further analysis. Sequencing files were aligned and annotated to the mouse genome (mm10) using the SPORTS1.1 pipeline^65^ with default parameters and a maximum number of mismatches of 2. Reference genome and small RNA annotation databases were downloaded from the SPORTS website (https://github.com/junchaoshi/sports1.1). This included the mm10 genome files, miRNA from miRbase 21, rRNA from National Center for Biotechnology Information (NCBI) Nucleotide, tRNA from GtRNAdb, piRNA from pirBase and piRNAbank, other ncRNA from ensembl (release-89), and rfam 12.3. The raw count tables generated by SPORTS were annotated to small RNA biotypes. Averages were aggregated across biotypes (rsRNA, tsRNA, miRNA, piRNA and so on) using the default annotations in SPORTS result output files.

The downstream analysis was carried out as previously described^66^ with few modifications. Briefly, counts were converted into reads per million (RPM). The fragments with at least 0.01 RPM in all of the samples and lengths between 16 and 45 nucleotides were retained for further analysis. Next, edgeR was used to identify differentially expressed fragments. All of the analyses were carried out using different packages in R version 4.1.2 and Bioconductor version 3.14.

#### RNA-seq library construction

Round spermatids, cauda spermatozoa and morula library construction and sequencing were outsourced to IGA Technology Services. Libraries were constructed using the Nextera Library Prep Kit (Illumina) according to the manufacturer’s instructions and sequenced on an Illumina HiSeq 2500 at 75-bp paired-ended (round spermatids and morula) or single-ended (cauda spermatozoa), with a minimum output of 40 million reads per sample.

Liver, muscle and epididymal white adipose tissue total RNA was prepared using TRIzol reagent (Thermo Fisher) according to the manufacturer’s instructions. RNA concentration and integrity were controlled on a Bioanalyzer system (Agilent) and only RNA samples with RIN values > 7 were used for downstream applications. Sequencing libraries were prepared by using the Quantseq 3′ mRNA-Seq mRNA Library Prep Kit FWD for Illumina (Lexogen) with i7 indexes (Lexogen) according to the manufacturer’s instructions. Libraries were sequenced on an Illumina HiSeq 2500 at 50-bp single-ended, with a minimum output of 40–50 million reads per sample.

#### RNA-seq data analysis

Reads mapping and differential expression analysis were carried out using the A.I.R. (Artificial Intelligence RNA-Seq) software from Sequentia Biotech with the following pipeline: BBDuk (reads trimming; BBDUkguide), STAR (reads mapping to the mouse genome GRCm38 (ENSEMBL); https://github.com/alexdobin/STAR), featureCounts (gene expression quantification; https://subread.sourceforge.net/featureCounts.html) and NOISeq (statistical analysis of differentially expressed genes; http://bioinfo.cipf.es/noiseq/doku.php). Compared to other methods to calculate differential expression, NOISeq is a data-adaptive non-parametric method specifically designed to account for high variability across replicates and genes with low expression levels^67^, a feature of RNA-seq datasets from both germ cells and developing embryos. Heat map and PCA analyses were carried out with the web application ClustVis using default parameters^68^ (Fig. 1c and Extended Data Figs. 1h, 2h, 6b,d and 8d,f) or with GraphPad Prism 8 or 9. Enrichment analyses were carried out with Enrichr^69^ or g:Profiler^70^ with default parameters.

For the heat map in Fig. 4f, plotted are manually annotated gene sets associated with multiple mitochondrial functions (see Supplementary Table 8 for the full list of genes). As most of these genes have tissue-specific expression, we plotted the average log_2_[FC(treated versus control)], for which the treated conditions are: direct HFD exposure for F_0_ tissues (gastrocnemius, epididymal white adipose tissue and sperm), paternal exposures for morula (HFD versus LFD; as we have only bulk RNA-seq data), and the phenotypic discordant HFD groups for both two-cell embryo (HFD_A versus HFD_B; see Fig. 3 and Extended Data Fig. 6 for the clustering) and adult F_1_ tissues (HFDi versus HFDt; see Fig. 1 for the group definition).

### Heteroplasmy experiment

#### Animals and husbandry conditions

A total of 60 female mice of the strain C57BL/6N-mt^ST^ (nuclear DNA: C57BL/6N; mtDNA: ST, GenBank accession number KC663621) of the age of 3 to 16 weeks were used. Mice were specific pathogen free according to FELASA recommendations and maintained in a barrier rodent facility. Groups of 3 to 4 females were housed in type IIL IVC cages (Blue Line, Tecniplast). The cages were lined with 120 g bedding (Lignocel Select, 3.5–4.5 mm poplar chips, Rettenmaier) and enriched with nesting material (PurZellin, Paul Hartmann) (photoperiod 12 h:12 h light/dark). Food (V1534, Ssniff Spezialdiaeten) and tap water in 250-ml bottles were available ad libitum.

These experiments were carried out at the University of Vienna (Austria) and all of the experimental procedures were discussed and approved by the Ethics and Welfare Committee of the University of Veterinary Medicine, Vienna and the national authority (Austrian Federal Ministry of Education, Science and Research) according to section 26ff of the Animal Experiments Act, Tierversuchsgesetz 2012–TVG 2012 under licence number 2021-0.731.149.

#### Experimental procedure

Female mice were treated in groups of 15 for superovulation by an intraperitoneal injection of 0.1 ml CARD HyperOva (CosmoBio, by Hölzel) and 48 h later 5 IU in 0.1 ml hCG (Chorulon; Intervet). Animals were euthanized 14 h after hCG injection, oviducts were dissected and ampullae were opened in drops of 90 μl HTF medium to collect the cumulus oocyte complexes. Oocytes were in vitro fertilized with sperm from LFD- or HFD-fed mice. Sperm from four different males per group were used as frozen–thawed sperm according to the EMMA protocol (https://www.infrafrontier.eu/). On each experimental date, oviducts from 7.5 females were used for IVF with a HFD male and oviducts from 7.5 females for IVF with LFD a male. On each date, different males were used. Briefly, thawed sperm was dissolved in 90 μl TYH medium and incubated for 30 min. A 10 μl volume of the sperm solution was added to a group of cumulus oocyte complexes and incubated for 4–6 h. After washing in HTF medium, oocytes were incubated overnight. Two-cell-stage embryos were washed the next morning in PBS, aliquoted in 0.2-ml tubes with lysis buffer^71^, snap-frozen in liquid nitrogen and stored at −80 °C.

#### Single-embryo RNA-seq and data analysis

A total of 122 HFD and 99 LFD single early two-cell embryos were processed to obtain RNA-seq libraries according to the Smart-seq2 protocol^71^ with 16 cycles of PCR pre-amplification. Libraries were verified using a 2100 Bioanalyzer (Agilent), pooled and paired-end sequenced on an Illumina NovaSeq 6000 platform (read length = 150 bp).

Data quality was assessed with MultiQC (version 1.11) and Nextera adaptors were removed from paired-end reads using trim_galore (version 0.6.6). Read mapping, gene expression quantification and differential expression analysis were carried out using the following pipeline: STAR (reads mapping to the mouse genome GRCm38 (ENSEMBL); https://github.com/alexdobin/STAR; after replacing the mtDNA sequence according to the strain C57BL/6N-mt^ST^; GenBank accession number KC663621); featureCounts (gene expression quantification; https://subread.sourceforge.net/featureCounts.html); DESeq2 (package 1.34.0; differential gene expression analysis). Gene Ontology (GO) enrichment analysis of differentially expressed genes has been carried out using g:Profiler^72^ with default parameters. Plotted are the driver GO terms from the ‘Molecular function’ and ‘Biological processes’ categories.

Individual embryos were sexed using the exclusive expression of Y-linked genes. For the analysis of mtDNA heteroplasmy, uniquely mapped reads were extracted from the BAM files using samtools and used to estimate mtDNA heteroplasmy with MitoHEAR (mitochondrial heteroplasmy analyzeR; https://github.com/ScialdoneLab/MitoHEAR)^73^.

Principal component and clustering analyses and visualization were carried out with the web application ClustVis^68^ with default parameters.

For Seurat-based clustering of the single embryos, the counts data matrix from featureCounts was used as input to the Seurat package to treat the individual sample as a single cell. The Seurat NormalizeData function was used to normalize counts. The highly variable genes were identified using the FindVariableFeatures function. The Seurat object was subsequently scaled using ScaleData and PCA was carried out using RunPCA. The FindNeighbors function was used to construct the nearest-neighbour graph with a dimensionality reduction of 1:15. Clusters were then identified using the FindClusters function with the resolution parameter of 0.8.

As one specific limitation of this method, smart-seq2 cannot detect fragmented tRNAs, as they are not poly-adenylated. To account for the partial penetrance of the reported phenotypes, we should have profiled sncRNAs in single embryos. Nevertheless, according to the genetic distance between the ST and the BL6 mtDNAs (which we used to calculate the heteroplasmy), only 5′ fragments of mt-Tp would be reliably detectable (which has an SNP at the 5′ and 3′ of the mature tRNA sequence). Therefore, although very likely, our data do not show transfer of mt-tsRNAs while demonstrating inheritance of paternal mature mt-tRNAs.

### Human studies

For the assessment of association of parental weight and the clinical phenotype of the offspring children, we analysed data from the Leipzig Childhood Obesity Cohort (NCT04491344) and the LIFE Child Study (NCT02550236), a prospective regional population-based longitudinal observational study aimed at characterizing contributing factors for civilization disease with comprehensive phenotyping (for example, clinical, laboratory, psychosocial data and biosamples) including data on parental BMI and BMI of the offspring conducted in the city of Leipzig, Germany^32,33^. With recruitment age ranging between the 24th week of gestation and 16 years of child age and annual follow-ups, the study combines a cross-sectional with a longitudinal design and covers a broad age range. The LIFE Child obesity sub-study specifically focuses on the origin and sequelae of childhood obesity in the frame of the Leipzig Childhood Obesity Cohort. After exclusion of children with present or past severe disease (for example, type 1 diabetes, syndromal obesity or cancer) and present or past interfering medical treatment (for example, insulin, immunosuppressives or growth hormone), we included those children with both parental BMIs available into the analysis (*n* = 3431; Supplementary Table 3). In the case of multiple visits, we used anthropometric data of the most recent visit of the child. For parental data, we used the earliest (that is, closest to the first pregnancy visit).

Expression of candidate genes derived from genome-wide expression data was analysed from children included in the Leipzig Adipose Tissue Childhood Cohort, for which subcutaneous adipose tissue samples and phenotypic data had been obtained as previously described^30^ (NCT02208141). RNA transcript levels were quantified using the Illumina HumanHT-12 v4.0 Expression BeadChip arrays and imputation, background correction and quality control were carried out according to previously published protocols^74^.

To correct for polygenic association of children’s BMI-SDS, we analysed 2,987 children of our cohorts for which genome-wide SNP array data, BMI-SDS and BMI of parents are available. Relatedness matrix was estimated according to ref. ^75^. The polygenic effect on BMI-SDS of children was subtracted by linear mixed model analysis using the R package GenABEL. We then tested the effects of parent BMI on BMI-SDS of children by linear regression analysis adjusting for age and sex.

#### sncRNA-seq from human spermatozoa

*Sample collection*. Samples were provided by our research collaborators from the University of Turku. After an informed consent, semen samples were given by 18 volunteering young men (19–21 years old). The men participated in a study on male reproductive health, which was approved by The Joint Ethics Committee of the University of Turku and Turku University Hospital. Ejaculation abstinence of at least 48 h was recommended before the semen sample collection. Standard semen analysis, including semen volume, pH, sperm concentration, total sperm counts and percentage of motile sperm, was carried out according to the World Health Organization Laboratory criteria in the Andrology laboratory of the Institute of Biomedicine. Spermatozoa were purified by centrifuging through a 50% gradient of Puresperm (Nidacon International). After washing the sample with PBS, the purity was evaluated microscopically, spermatozoa were counted, and the sample was evaluated for somatic cell contamination. The pellet was resuspended in Sperm CryoProtec II (Nidacon International AB) and frozen pellet was shipped to Germany. In Germany, spermatozoa were further purified from somatic cell contamination by washing with somatic cell lysis buffer^76^. The purity of samples was validated microscopically. Detailed sample information including sperm and metabolic parameters can be found in Supplementary Table 4.

RNA isolation. Total RNA was extracted from spermatozoa with the TRIzol–chloroform phase separation method followed by precipitation and wash steps with 100% and 70% ethanol, respectively. RNA quality control was carried out with Agilent Bioanalyzer and only the samples with a RIN value of between 2 and 4.5 and no evident intact ribosomal RNA peaks were further processed.

Library preparation. Sequencing libraries were prepared using NEBNext Small RNA Library Prep Set for Illumina (New England Biolabs) with 100–120 ng RNA as a total input. To minimize adaptor-dimer formation, adaptors were diluted 1:6, whereas reverse transcription primer was applied undiluted. At the final PCR stage, 1:2 diluted primers were used. Amplified libraries were cleaned with Monarch PCR & DNA Cleanup Kit (New England Biolabs) and size selected with AMPure XP (Beckman Coulter) using 1.0× and 3.7× beads for long-fragment removal and target-size retention, respectively. Pooled libraries were sequenced on a NovaSeq 6000, SP flow cell, 100 cycles (Illumina) at an average depth of 53.18 million reads per sample.

Data analysis. All sequencing files were quality checked using MultiQC v1.11. After a preliminary quality check, single-end read sequences were analysed using SPORTS1.1 (ref. ^65^). The adaptor sequence (5′-AGATCGGAAGAGCACACGTCTGAACTCCAGTCA-3′) was trimmed using in-built Cutadapt pipeline and sequences with the length of 15–45 nucleotides were further processed. Using human genome version hg19 and allowed mismatch number set to 2, SPORTS1.1 mapped reads to small RNA subtypes. After joining sequences from all samples into a common count matrix, a filter was applied to exclude sequences with no reads in more than 80% of samples. Samples were further analysed on the basis of BMI variation. Sequence-based differential expression analysis was carried out using DESeq2 (ref. ^77^) addressing BMI as a continuous variable. Briefly, a generalized linear model-based algorithm estimated the association of each sequence with the BMI across samples and output log_2_[FC] reflecting change in sequence expression level per unit of increment of BMI. Benjamini–Hochberg *P*_adj_ < 0.1 served as a measure of significance for each result. Pearson-based correlation analysis between donors’ BMI and sperm sncRNA biotypes (variance-stabilizing transformation-normalized expression) has been carried out with GraphPad Prism 8 using default parameters.

The following string represents the code used for the continuous DESeq2 analysis of human sperm sncRNA-seq data: dd_obj <- DESeqDataSetFromMatrix(countData = [your sequence count matrix], colData = [data frame with sample ID and BMI values], design = ~BMI).

### International Mouse Phenotyping Consortium data collection and analysis

The goal is to provide evidence in support of the hypothesis that paternal mitochondrial dysfunction leads to intergenerational alterations of metabolic homeostasis. The IMPC offers an invaluable resource of functional genetics studies^29^. Briefly, we used the IMPC dataset to study the (epi)genetic, intergenerational consequences of paternal manipulation of genes involved in mitochondrial structure and function.

For this, we compared a battery of 11 numerical metabolic phenotypes (fat/body weight; initial response to intraperitoneal glucose load; AUC_ipGTT_; total food intake; respiratory exchange ratio; total cholesterol; HDL cholesterol; triglycerides; fasting blood glucose; albumin; alkaline phosphatase) in two populations of isogenic WT C57BL/6N mice generated from a pure WT lineage (control) or from heterozygous matings (WT—parental information (father × mother): het_×_het; het_×_WT; WT_×_het).

#### Gene selection

The selection of genes involved in mitochondria structure and function has been carried out with the following steps: from the list of IMPC genes (Data Release 11), we extracted those presenting with one or more of the above listed metabolic phenotypes in heterozygosity; functional enrichment analysis (GO and KEGG pathway analysis using Enrichr^69^); list of selected genes compiled by merging genes belonging to the following mt-related terms (GO:0005739; GO:0005743; GO:0005759; GO:0031966; GO:0042645; GO:0005747; GO:0005761; GO:0005763; GO:0005749; GO:0032592; KEGG:mmu00190; Supplementary Table 5).

#### Data collection and analysis

As the individual IMPC phenotyping centres phenotype control and/or WT animals (see definition above) and consider them as WT control for gene-specific phenotypes, specific control or WT data are not directly identifiable from the IMPC website. Therefore, we were granted special access to the IMPC data for specific collection of control and WT phenotypic information. Following collection, the dataset was arranged in a multidimensional data matrix for further analysis. Animals with at least three replicates per group and sex were included for further steps. Imputation for the missing data was carried out with a random forest imputation algorithm using the missForestR package with default parameters^78^. The data matrix was then scaled and the prcomp function in R was used to determine the principal components of the dataset. To quantify the difference between the genes on the basis of phenotypes, we use the Pearson correlation method using the get_dist function from the factoextra R package. These correlation coefficients were calculated to identify similarity patterns in gene–phenotype pairs and visualized in a heat map generated by using the ComplexHeatmap package from R. Cluster-specific phenotypes were visualized in a heat map including the 11 phenotypes, as well as sex- and parent-of-origin-specific information. Ranked AUC_ipGTT_ (expressed as log_2_[FC(WT versus control)]) was plotted as a horizontal bar plot using GraphPad Prism 9.

#### Cryopreserved sperm collection

Cryopreserved sperm samples containing a pool of purified cauda spermatozoa from 10 heterozygous mice were obtained from EMMA^56^. Genes were selected for availability with the exception of *Tsfm* (Ts translation elongation factor, mitochondrial), which represents a suitable negative control for initial mechanistic dissection.

#### RNA isolation and sncRNA-seq libraries construction

Individual straws were thawed directly in TRIzol and RNA was extracted with the miRNeasy Micro Kit (QIAGEN 1071023) according to the manufacturer’s instructions. A 10 ng quantity of total RNA was used for sncRNA library preparation. Libraries were prepared using the NEBNext Multiplex Small RNA Library Prep Set for Illumina (NEB E7560S) with 5′ and 3′ adaptors diluted 1:5 and 15 PCR amplification cycles. Libraries were verified using a 2100 Bioanalyzer (Agilent) and paired-end (read length = 150 bp) sequenced with the Illumina NovaSeq 6000 platform.

#### sncRNA-seq data analysis

sncRNA-seq data were analysed as described above. Briefly, sequencing raw data were quality checked using MultiQC v1.11. Reads were trimmed using cutadapt 2.8 according to the kit manufacturer instructions. Trimmed and quality-filtered reads were used for further analysis. Sequencing files were aligned and annotated to the mouse genome (mm10) using the SPORTS1.1 pipeline^65^ with default parameters and a maximum number of mismatch of 2. Reference genome and small RNA annotation databases were downloaded from the SPORTS website (https://github.com/junchaoshi/sports1.1). This included the mm10 genome files, miRNA from miRbase 21, rRNA from NCBI Nucleotide, tRNA from GtRNAdb, piRNA from pirBase and piRNAbank, other ncRNA from ensembl (release-89), and rfam 12.3. The raw count tables generated by SPORTS were annotated to small RNA biotypes. Averages were aggregated across biotypes (rsRNA, tsRNA, miRNA, piRNA and so on) using the default annotations in SPORTS result output files.

### Statistical analysis

All figures and statistical analyses (as needed and appropriate) were generated using GraphPad Prism 8 or 9. Statistical significance was tested by Student’s *t*-test, or ANOVA as appropriate. Correlation analyses were used to test for linear regression. Odds ratios have been calculated using MedCalc (https://www.medcalc.org/calc/odds_ratio.php). All data are expressed as mean ± s.e.m. and a two-tailed *P* value < 0.05 was considered to indicate statistical significance unless otherwise specified in the text.

### Reporting summary

Further information on research design is available in the Nature Portfolio Reporting Summary linked to this article.

## Online content

Any methods, additional references, Nature Portfolio reporting summaries, source data, extended data, supplementary information, acknowledgements, peer review information; details of author contributions and competing interests; and statements of data and code availability are available at 10.1038/s41586-024-07472-3.

### Supplementary information

Reporting Summary

Supplementary Tables Supplementary Tables 1–8.

### Source data

Source Data Fig. 1 and Extended Data Figs. 1 and 2.

## Data Availability

All of the raw sequencing data have been deposited at the NCBI Gene Expression Omnibus under accession number GSE239815. Processed sequencing data and anonymized human data will be provided by the corresponding author upon reasonable request. Source data are provided with this paper.
